# Eating Behaviors of Children with Autism—Pilot Study, Part II

**DOI:** 10.3390/nu13113850

**Published:** 2021-10-28

**Authors:** Beata Kazek, Anna Brzóska, Justyna Paprocka, Tomasz Iwanicki, Karolina Kozioł, Agnieszka Kapinos-Gorczyca, Wirginia Likus, Małgorzata Ferlewicz, Agnieszka Babraj, Agata Buczek, Irena Krupka-Matuszczyk, Ewa Emich-Widera

**Affiliations:** 1 Child Development Support Center “Persevere”, 40-583 Katowice, Poland; beakazek@op.pl (B.K.); annabrzoska@poczta.onet.pl (A.B.); malgorzata.m.ferlewicz@gmail.com (M.F.); agabialowas@wp.pl (A.B.); 2 Department of Pediatric Neurology, Faculty of Medical Sciences, Medical University of Silesia, 40-583 Katowice, Poland; koziolkarola@gmail.com (K.K.); marekwidera@wp.pl (E.E.-W.); 3 Department of Biochemistry and Medical Genetics, School of Health Sciences in Katowice, Medical University of Silesia in Katowice, Medyków Street 18, 40-752 Katowice, Poland; t.iwanicki@sum.edu.pl; 4 Department of Pediatrics and Pediatric Endocrinology, Faculty of Medical Sciences, Medical University of Silesia, 40-583 Katowice, Poland; 5 CZP Feniks, Daily Ward for Children and Adolescents, Młyńska 8, 44-100 Gliwice, Poland; aga27.11@interia.eu; 6 Department of Anatomy, School of Health Sciences, Medical University of Silesia, 40-752 Katowice, Poland; wirginia.likus@gmail.com; 7 Department of Neurological Rehabilitation, John Paul II Upper Silesian Child Health Centre, Teaching Hospital No. 6 in Katowice, Medyków Street, 40-583 Katowice, Poland; neurologopedaagatabuczek@gmail.com; 8 Higher Medical School in Silesia, Mickiewicz Street 29, 40-085 Katowice, Poland; irena@matuszczyk.pl

**Keywords:** ASD—autism spectrum disorder, ADOS-2 autism diagnostic observation schedule second edition, ADOS-2

## Abstract

Autism spectrum disorder is characterized by social communication deficit and non-normative behavior. The people with autism often experience troubles with feeding. The purpose of this study was to conduct evaluation of the feeding and eating behaviors among children with autism. Patients and Methods: The study group included 41 high-functioning autistic children. The control group consisted of 34 children without the ASD. The questionnaire was used to assess the nutritional status. Results: The children with ASD fuss during mealtimes more frequently, they require entertaining and diverting their attention, they are fed by parents, and they consume their meals away from the table. The significant difference found in the use of utensils and food selectivity works to the disadvantage of the Study Group. Conclusions: The food selectivity occurs significantly more frequently among children with ASD. The feeding and eating problems should be considered on a wider scale. The cooperation of the multidisciplinary and the parents teams should be proposed in the ASD patients care.

## 1. Introduction

Autism spectrum disorder (ASD) is a pervasive developmental disorder which is characterized by social communication deficit and non-normative behavior (ICD–10, DSM 5). About 1.5% of children in the developing stage suffer from autism spectrum disorder (data as of 2014 according to the CDC, 1 in 68 children) [1].

The etiology of autism remains unknown, but genetic factors along with triggering environmental factors are thought to play the primary roles [2,3,4].

Autistic people experience many comorbid health problems. Gastrointestinal disorders are among the most common somatic disorders observed in 46–91% of patients in comparison to 6–50% of healthy peers. Even more distinct differences are found in feeding [5,6,7,8,9,10,11].

Feeding/eating problems are observed in 10–20% of all children (with exacerbation in children under 3 years of age). This problem is reported by parents of at least 70% of autistic children [5,7,12,13,14,15].

Feeding/eating problems may be the result of:

- Biological factors (including somatic factors such as allergies, food intolerance, gastrointestinal disorders, gastrointestinal motility disorders--Including GERD, constipation, and diarrhea—Inflammation in the gastrointestinal tract, changes in the mouth—dentition state and inflammation—and fear of unpleasant difficulties.
- Neurodevelopmental factors such as skill-based deficits, oral motor problems, difficulty chewing and swallowing, sensorimotor disorders, and attention switching.
- Social, relational and/or behavioral factors (maladaptive mealtime behavior) [16].

The most frequent eating problems occurring among children with ASD and their healthy peers are similar; some researchers agree that the issues with feeding that are developmentally appropriate amplify in the ASD patients [17]. According to Brede, there are certain characteristics of ASD that may promote restrictive eating problems, and they include sensory sensitivities, social interaction and relationship difficulties, sense of self and identity issues, difficulties with emotions, autistic thinking styles, need for control and predictability (impact-direct and indirect) [3].

The coexistence of psychiatric disorders are characteristic of people with autism. Some of these disorders may also impact feeding patterns. The most common disorders that coincide with ASD in the developmental stage are the following: Attention Deficit Hyperactivity Disorder (ADHD; 55%); anxiety disorders, including specific phobias occurring in 30% of patients; obsessive compulsive disorder, occurring in 17% of patients; and depression, occurring in 38% of patients [18].

Children with ASD, in addition to suffering from OCD, experience sleep and appetite disturbances, as well as peculiar eating patterns which may be characterized as “eating and running”, “no time to eat” or “easily distracted” [19,20].

Seventy to eighty percent of children with ASD suffer from a sensory processing disorder which significantly affects social communication and behavior, and is further correlated with sensory viewing of the world, motor skills, posture, praxis, and eating behavior [19,21].

In recent years, attention has been paid to the intestine’s role as the “second” brain, due to the existence of nearly 100 million neurons in this organ and the activity of the gut–brain axis facilitating the two-way hormonal, nervous, immunological and metabolic communication. Functioning of the gut–brain axis has also been considered in a few theories on the development of the autism spectrum disorder [21,22,23] as well as in nutritional therapy trials (dietary treatment). Dietary interventions, including mostly dairy-free and gluten-free diets, are introduced for 25% of autistic children. These diets are enhanced with supplements and vitamins [24].

Due to the fact that, for children with autism, ways of eating and theconsequences thereof have still not been fully understood and elucidated, the purpose of the study was to analyze the eating behaviors of children with autism, including the process of shaping eating habits, everyday eating patterns of the applied diet, and the occurrence of food selectivity.

## 2. Materials and Methods

**Figure 1:**
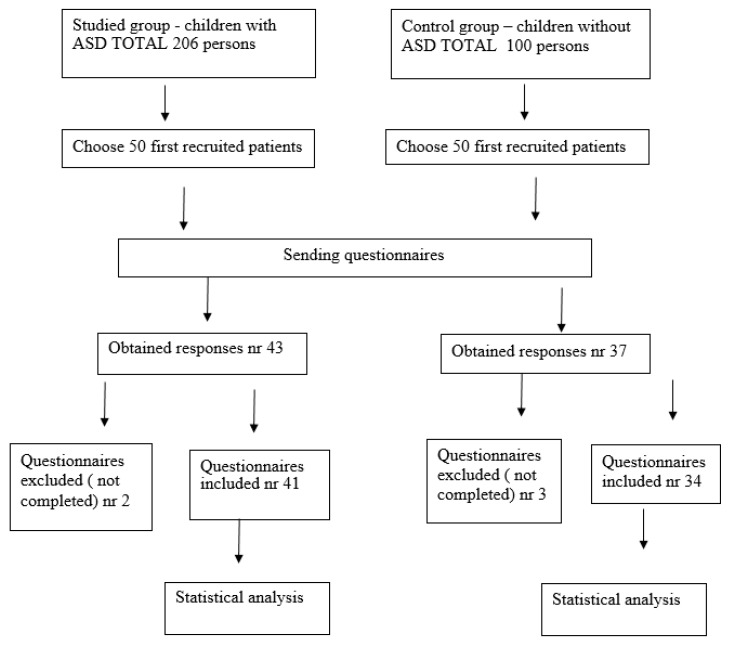
Study Flow Diagram.

The study materials included two groups of children: the study group of 41 autistic children and the control group of 34 healthy children [Figure 1].

### 2.1. The Characteristic of the Studied Group

The study was performed among the autistic Caucasian children under the care of the Neurology Clinic of the Child Development Support Center “Persevere” in Katowice, the Child and Youth Outpatient Mental Health Clinic “NZOZ Feniks” in Katowice, and the Neurology Outpatient Clinic at John Paul II Upper Silesian Child Health Centre in Katowice. In selecting the participants for the studied group, the following inclusion and exclusion criteria have been applied:

Inclusion

- Informed and voluntary consent of the child’s parent/legal guardian to take part in the study.
- Children between 2 and 12 years old.
- Diagnosis made by a psychiatrist (using the gold-standard diagnostic test—ADOS-2).

Exclusion

- Syndromic autism (additionally diagnosed mental disorder, epilepsy, congenital disorder, or genetic disorder).
- IQ < 70.

### 2.2. Characteristics of the Control Group

Caucasian children who have been the patients of pediatric clinics in the cities of Tychy and Katowice were recruited to the control group. The following inclusion and exclusion criteria were applied in selecting the participants for the control group:

Inclusion

- Informed and voluntary consent of the child’s parent/legal guardian to take part in the study.
- Children between 2 and 12 years old.
- Absence of pervasive developmental disorder (as confirmed by a child and an adolescent psychiatrist, a child neurologist, a psychologist and an educational research scientist).
- Absence of any diagnosis of chronic conditions that could potentially affect the nutritional status (previously diagnosed digestive tract diseases).

Exclusion

- Children younger than 2 and older than 12 years.
- IQ < 70.

### 2.3. Study Method

The parents were informed about the voluntary and anonymous character of participation in the study and its solely scientific purpose. The paper version of the questionnaire was filled out by the child’s caregiver at home, without either the presence of the interviewer or time restrictions. The study was conducted based on the diagnostic survey. The questionnaire, of our own design and developed for the purpose of conducting a detailed nutritional assessment considered on many levels, was utilized as the study tool. The first part of the questionnaire contained information regarding the child’s gender, date of birth, and the date of filling out the questionnaire. The questionnaire included two parts. Part I had 20 questions and related to the nutritional status of the child in the first year of life as well as the nutritional status of the mother during breastfeeding. The questions referred to the following: method of feeding the child in the initial period of life (breastfeeding or formula); method of introducing complimentary foods; and any difficulties while introducing changes in the feeding. (The results have been published: Brzóska A. et al. Eating Behaviors of Children with Autism–Pilot Study [25]).

Part II consisted of 20 questions, related to the current nutritional status of the child in terms of the child’s eating and feeding behavior, habits, and preferences. An additional part of the questionnaire was an Attachment with 12 questions which related to not only the child’s eating and feeding habits but also to the eating habits of the child’s family. Most of the questions of the questionnaire were closed-ended questions where the respondent would have to choose between the YES (Y) or NO (N) answers to answer the particular question. There were also multiple choice questions where the respondent would mark the proper answer with (X). The remaining few questions were open questions to which the respondent would provide a descriptive answer.

### 2.4. Statistical Methods

Many statistical hypotheses have been verified as part of the statistical analysis of the study results. The Chi-Square Test for Association with Yates continuity correction was used in the multiple-field tables, and the Fisher Exact Probability Test was used for the variables in the nominal scale and presented in the four-field tables.

While performing the calculations, we concluded that the negative answers provided for the closed-ended questions were also negative in relation to the variants of the answers proposed in those questions.

For the variables in the ordinal scale (age and grade scale of food/product choice), we used the nonparametric Mann-Whitney U Test in the statistical assessment of the differences in the results of both groups. The test was performed due to the significantly statistically different distributions of the analyzed quantities from the theoretically normal distribution. (Shapiro–Wilk Test) All statistical hypotheses have been verified by applying the standard variable value answers proposed.

## 3. Results

Both groups were homogeneous in terms of sex (*p* = 0.3) (Table 1) and age (*p* = 0.73).

The “Results” chapter has been divided into smaller parts in accordance with the format used in the questionnaire included in the attachment to the paper.

### 3.1. Questionnaire—Part II The Child’s Current Nutritional Status (20 Questions)

There was no statistically significant difference between the groups in terms of duration of meals as well as attitude towards the meals consumed (with pleasure, indifferent attitude towards food, or being forced to eat), but the approach of forcing the child to eat occurred in 11% of one control group and 29% of the other (Table 2). The children of both groups did not consume any meals at night and the groups also did not differ in terms of the number of consumed snacks during the day.

Question No. 5 asks the respondents about the type of snacks the children consume during the day. The results of the statistical analysis in this respect did not show any statistically significant differences between the groups for any of the suggested types of snacks (fruit, vegetables, bread, sweets, and salty snacks).

In terms of the children’s current appetite in both groups (proper, increased, reduced, loss of appetite), decreased appetite was found to occur significantly more frequently in the study group (understood as reduced appetite and loss of appetite) (Table 3).

The result of the statistical analysis regarding the answer to the question of whether the child fusses during mealtime showed a clear difference between the groups. Fussing during mealtime occurred twice more frequently with children in the study group than with children in the control group (Table 4).

The answer to the question of whether or not a child requires entertaining or diverting of attention during mealtime was also consistent with expectations; the groups differ significantly in that the necessity of providing entertainment was nearly five times greater for the children in the study group compared to the children in the control group (question II/8, Table 5).

The answers to the question of whether or not the child consumes meals together with the other members of the family did not show any statistically significant difference between the groups.

Similarly, for question No. 10 of Part II, concerning the regularity of consumed meals the results also did not show any statistically significant difference between the groups.

However, for question 11, concerning the child’s position while consuming meals (with the proposed answers being: sitting at the table, standing, walking to the table, or sitting on the floor), the results of the statistical analysis showed a statistically significant difference in the study group, for meals consumed away from the table, while sitting on the floor (Table 6).

Results of the statistical analysis regarding the answer to question 12, concerningthe way the child consumes meals, were gathered in Table 7. A significant difference was found between the groups in relation to the less frequent use of utensils by the children of the study group, where the use of spoons was *p* = 0.02, and the use of forks was *p* = 0.001, while being fed by the caregiver four times more frequently *p* = 0.0004 (Table 7).

A statistically significant difference was found between the groups regarding food selectivity which was twice more frequently observed in the study group than in the control group (question II/13, Table 8), although the selectivity did not relate to any specific group of food products (question II/13).

While formulating question 14, a range of tastes were suggested that could be preferred by the children in both groups. The results of the analysis in this regard were gathered in Table 9. The only statistically significant difference found related to the answer “a. sweet” and referred to the healthy children from the control group.

The results of the statistical analysis regarding the variables of the answers to question 15 referring to the texture of the consumed foods preferred by the child were also collected, and it was found that the children from the study group clearly prefer the fluid texture (Table 10).

The answers to the question on the preferred temperature of the consumed meal (optimal temperature and temperature less than optimal) as well as its fragrance however, did not indicate any statistically significant difference.

The parents in both groups were asked to grade the food preferences of their children. The following food products were listed to choose from: milk and dairy products, meat, eggs, fish, vegetables, fruit, and processed food. The differences, between the groups, in responses about the proposed foods did not result in any statistical significance.

Among the answers to the question on who the decision maker is in choosing the meals consumed by the child (parent/caregiver or the child), the answer referring to the child indicated the statistical significance (Table 11).

The last question, No. 20 of part II of the questionnaire and which was a closed-ended question, referred to the use of a restrictive diet. While a positive reply was given by 17% of the study group and 6% of the control group, a statistical significance was not indicated.

The respondents who provided positive answers to the closed-ended part of question No. 20 also provided their comments on the proposed types of restrictive diets (dairy-free, lactose-free, gluten-free, egg-free, hypoallergenic, candida, and other). The results of the statistical analysis in that respect were gathered in Table 12 showing that the groups differ significantly only in terms of answer a., dairy-free type of diet (Table 12).

### 3.2. Questionnaire–Attachment–Additional Questionnaire on the Assessment of the Eating Behaviors—12 Questions

The first question in the attached questionnaire asked the respondents about the characteristics of the meals/food products the children in both groups prefer. It was aimed at providing the degree of importance of the specific characteristics (from 0 to 10) of the food preferred by the child. A statistically significant difference was not found in terms of color, shape, fragrance, or taste of the food.

In terms of food texture, a statistically significant difference was found (*p* = 0.003). At the same time, it is worth pointing out the relatively low degree of importance indicated for the texture of foods by the control group (Table 13).

The groups did not differ statistically on the subject of tasting new foods. Similarly, a significant difference was not found between the groups in terms of the age the child was when the parent caring for the child returned to work. While the results did show that parents caring for the children in the study group returned to work earlier than the parents of the children from the control group, this difference is also not statistically significant.

Furthermore, the result of the Chi-Square test c oncerning the answers, from the two groups, on who stayed with the child when both parents returned to work (close family members, nanny, caregivers at daycare center, or caregivers at preschool) did not indicate any significant difference. A statistically significant difference was also not found in the answers to the question of whether the family owned a table in the kitchen. Furthermore, the number of family mealtimes during the week was similar in both groups and the answers concerning the type of meal consumed together with the child also showed no statistically significant difference.

When asking the question regarding the method of convincing the child to eat, 11 answer choices were proposed. Eight of these answers (except for answers c, e, and f) did not show any statistically significant difference. The majority of the parents in the study group used the sentence “if you eat, you will get/go…” (*p* = 0.03) but, at the same time, various methods of presenting food were utilized significantly more frequently in this group (*p* = 0.05). “Family mealtimes” was utilized as motivation significantly more frequently in the control group (*p* = 0.01) (Table 14).

The result of the test conducted to assess the difference between the groups in terms of whether or not the child consumes meals more willingly when the whole family is sitting at the table indicates the significance of the social aspect of family mealtimes in the families of the healthy children (*p* = 0.04; Table 15).

The self-efficacy scale in question 11 allowed us to determine that the frequency of parents using different devices, such as radio, television, or reading newspapers, etc., during mealtimes is significantly higher in the study group (Table 16), specifically in the high ranges of 5 to 10, on a scale of 1 to 10, being six times more frequent (Table 17).

The last question, No. 12, included in the Attachment to the questionnaire, asked the Respondents to indicate whether there is any particular meal that is especially important to all members of the family. Both groups were unequivocally comparable (absence of statistical significance). The meals indicated by the parents of both groups to be especially important to all members of the family included pizza, spaghetti, and chicken broth with noodles, as well as the traditional holiday meals and birthday cake.

## 4. Discussion

Children’s eating behaviors develop throughout the entire period of development, starting from the prenatal period. Eating behavior patterns are influenced by genetic predisposition, family and its eating behaviors, educational institutions, peer groups, and mass media. Due to the axis problems as well as comorbid disorders, children with ASD exhibit very different eating behaviors.

Based on the conducted questionnaire-based investigation, the analysis of the behaviors, habits, and food preferences of the children in the study group in comparison to the group of healthy peers was undertaken, taking the infant [25], early childhood, and school periods into consideration. The period between the thirteenth and thirty-sixth month is critical for the development of proper eating behaviors, habits, and preferences as cognitive curiosity is also directed towards eating and the rituals related to it, while the child learns about food products through its senses. This period of life is also about significant criticism of the new and the unknown, which bears a risk of developing an interim state of food neophobia. The parent role is to show patience and consistency in presenting the child with new foods, fragrances, and tastes because, as the research studies have shown, the acceptance of the “new” may require even up to 15 trials. It is important in this period that parents present the child with healthy behaviors related to food while creating a peaceful and pleasant feeling around it [26].

The appetite, in general, was described as “reduced” by the mothers of the autistic children. Based on the professional literature and our own clinical experience, we think that the connection between reduced appetite and problems related to food selectivity, sensory disorders, and gastrointestinal disorders seems well-founded [5,6,7,21,27].

When comparing the environment and the circumstances revolving around consuming meals by both groups, it was observed that most of the healthy children consume meals with pleasure, while the autistic children eat under pressure. Every third autistic child and every tenth healthy child is forced to eat. The problem of eating and feeding concerning autistic children is a great emotional burden, especially among the families of autistic children [28]. The result of feeding the autistic child is stress, which is related not only to the feeding process itself but also to its consequences. The parents of the autistic children are worried that the feeding problem may lead to undernutrition and they therefore strive to intensify efficiency of their actions. At the same time, it is observed that direct commands (by feeding parents) are related to better results of feeding, which then justifies and enhances the frequently adopted directive attitude of the feeding caregiver [29].

Nevertheless, the cause of this phenomenon requires further investigation on an individual level of each patient. We need to consider the behavioral and social grounds as well as to remember that the gastrointestinal disorders occur among autistic children much more frequently than among healthy peers, and that the children with gastrointestinal tract diseases are more likely to develop feeding and eating problems [11,30].

It is rather unsettling in terms of health that autistic children consume meals with the use of the high technology devices twice more frequently than their healthy peers. Research studies show that parents willingly and frequently attract the child’s attention to the screen during mealtime, or when the child is falling asleep, crying, or playing. When the child only seems to calm down in front of the screen, becoming still and not responding to any other stimuli, the parents perceivethat as a positive outcome, allowing them to “effectively” feed the child. However, high technology devices shape the learning model which is based on “flat screen”, without the physical, emotional, or sensory contact in the form of touch, fragrance, or taste. While consuming a meal in front of a TV, laptop, or phone, the child does not pay any attention to the taste, fragrance, or appearance of the food or its quantity; the motivation to eat it is watching a cartoon or playing a computer game without the feeling of hunger, which may consequently aggravate the feeding and eating problems as well as the developmental problems [31,32].

The results of the self-efficacy assessment indicate that the parents of the children with ASD use the high technology devices themselves during family mealtimes, with increased frequency (5–10 on a scale of 0 to 10). It has also been observed by other researchers that the screen behaviors of parents are associated with higher viewing of TV by the children [32].

This finding is all the more concerning because it was also found that screen time during mealtime and unhealthy feeding behaviors are profoundly connected with each other, especially in early childhood [33]. In addition to that, the excessive screen time (>60 min daily) used by young children may be associated with antisocial and aggressive behaviors, problems with sleep, the deficits in psychomotor development, and overweight/obesity [32].

Our own research shows that autistic children are fed by the parents four times more frequently (Table 7), while healthy children consume meals independently, using utensils. Such behaviors may in fact be resulting from the substantially reduced appetite among autistic children (Table 3) as well as the fine motor skills disorder quite frequently occurring among this group of children, making it difficult to use utensils efficiently [15,34]. The research study of Stough et al. points out the significantly higher efficiency of the parent feeding the child which may explain the significantly greater frequency of feeding by the parents in the study group (Table 7) [29]. The average time of meal consumption in both groups was between 10 to 20 min, which is consistent with the principles of proper feeding. The eating behavior pattern observed in the autistic children based on this analysis shows how complex and difficult the problem of feeding is to the parents/caregivers, which is confirmed in the available literature [13,35,36].

The autistic children and the healthy children consumed meals together with the other family members as much as it is possible. The majority of the parents from both groups declared that they possess a table in the kitchen or the dining room. The statistically significant difference was not found between the families with the children with ASD and the families with the healthy children in terms of the number of family mealtimes spent at the table on a weekly basis. The average number of family mealtimes was 13, which is approximately two family mealtimes daily. We have also attempted to establish what type of meal is consumed most often together with the child. Both groups indicated breakfast and lunch. Supper, however, is usually consumed together in families of the children without any developmental disorders. Family mealtimes is a confirmed incentive to eating, for the healthy children, while this correlation was not observed in the autistic children. Individuals with autism do not adjust their eating behaviors to other people. Moreover, eating together with others may trigger sensory overstimulation (fragrances, sounds, or visual stimulation) which may manifest as frequent exacerbation of difficult behaviors in the presence of other people eating. Consuming meals with others is not only for the purpose of eating but also for talking and being together at the table. The autistic child may have a problem with undertaking all those activities at the same time. On the contrary, it may be more pleasant for autistic children to consume meals on their own. (Table 5 and Table 15) [29].

The children of both groups consume meals while sitting at the table, but the Respondents of the study group indicated the option of walking to the table or eating while moving slightly more often. This result, though, did not indicate any statistical significance. Furthermore, 10% of children from the study group eat while sitting on the floor which, due to the absence of such behaviors in the healthy children, indicated statistical significance. Other authors also report the difficulties in remaining in the sitting position at the table, leaving the table, and eating in other locations than the table [37].

The results of this study show that the decision maker in terms of choosing the type of meals, in the control group, is the parent while, in the study group, the decision is dependent on the child’s capabilities. Due to the difficulties related to feeding, although the parents decide about the preparation of the meals, the success of the child eating the meal is not guaranteed. The children of the control group in the age above 1 year do not, for the most part, have restrictive diets. In the analysis of the subgroup being on diets, though, the tests indicated statistically significant differences. Based on the gathered data, two diets most often introduced among the autistic children include the dairy-free (statistically significant) [38] and gluten-free diets. These observations are consistent with the results of other scientific research studies [5,11]. The fact that nutritionists are not involved in the nutrition of the children with ASD is unsettling. We may, therefore, conclude that the parents of the children with this disorder make the decision themselves to introduce individual diets for their child which bears a risk of negative consequences for the child’s health in the form of eating disorders or insufficient supplementation of nutrients. The poor nutritional status and malnutrition of children with autism have been the subject matter of research studies and constitute a serious problem in the child’s development, with the risk of adverse outcomes in adult life [15,39]. The analysis conducted in this study does not indicate any differences in the everyday practice of feeding the autistic children and the children without any developmental disorders. The parents of both groups report to provide regular consumption of meals by their children. The number of snacks, understood as the food products consumed between the main meals, was assessed in both groups as two, and the most frequently chosen snacks included sweets and fruit. Currently, attention is paid to the problem of consuming too much of high-calorie foods by children. Sweets being the dominant snack show this problem.

Food selectivity is very often considered by parents and caregivers in terms of the following factors: texture (69%), general appearance (58%), taste (45%), smell 36% and temperature (22%) [5]. The food preferences assessed in this study were evaluated by asking two independent questions. The food preferences of the children with autism were analyzed in terms of the degree of importance of the particular characteristic for the child. We assessed the attitude of the child towards color, shape, fragrances, taste, and texture. Significant differences were indicated for the texture of food, where the importance for this characteristic was assessed at 5 in a 10-degree scale for the children with autism, while the same was assessed at only 2 for the healthy children (Table 13). The preferences were also assessed in terms of taste, fragrance, texture, and temperature with a closed-ended question, and the statistical significance was in the autistic children choosing fluids most willingly (Table 10). The results are consistent with the observations of other researchers who indicated that texture is the most frequent factor (up to 69%) the autistic children consider in food [28]. Such preferences may derive from the sensory processing disorder (the simultaneous processing of information, arriving through many senses, during the act of eating). Oral health problems, such as dental damage and a possible inability to chew, may also affect the preferences [35]. The need of “sameness” cannot be excluded as the influencing factor in selecting food by autistic individuals. Skill-based problems with eating, such as problems with swallowing, reported by the parents of the autistic children have an undeniable effect on the selection of food [15,28,34,40]. The clear preference of fluids may also be an indication of inaccurate interpretation of hunger as thirst, which could also be an outcome of the problems with self-regulation. Nevertheless, this thesis has not been confirmed by any studies and requires further research.

In eating, both, children and adults, consider taste, fragrance, color, or texture of food, and that is how people create their food preferences. If any characteristic of food becomes such a restrictive criterion for a child that it consequently limits the acceptable list of food just to a few products, then attention must be paid to the problem of food selectivity. Establishing the cause may help with indicating the therapeutic method [28,35,41].

From the opinion of the parents, both the healthy children and the autistic children do not like fish and vegetables, while sweets were not indicated as a “disliked product” at all. The scientific reasoning for the abovementioned observations is the innate preference of the sweet taste along with the lack of acceptance of the bitter taste. Fruit and sweets that have a dominant sweet taste are, therefore, most frequently chosen for snacking, and vegetables with the predominantly bitter taste are avoided by children [28]. Although the children from both groups gladly choose the sweet snacks, the significant preference of the sweet taste was, surprisingly, indicated among the healthy children. Perhaps, this may result from the better nutritional awareness of the parents of the autistic children and their decision to restrict the sugar intake early in the lives of their children; nevertheless, this requires further studies and analyses (Table 9).

This study confirms that food selectivity occurs significantly more frequently among the children with diagnosed autistic spectrum disorder. Based on the conducted analysis, we cannot indicate just one group of foods preferred by the majority of the autistic children. However, the analysis shows that food selectivity includes the products from the “milk and dairy products” category the least frequently. These results are consistent with studies by other researchers on feeding and eating problems [13,42]. The tendency of the children with the autistic spectrum disorder may be the result of problems with the functioning of the gut–brain axis, which fits the opioidergic theory on the autistic disorder [5]. Furthermore, according to some researchers, casein has been considered to be the etiopathogenetic factor of autism [38].

Food neophobia, or reluctance to try new and unknown products, is rooted in genetics as well as the environment, and it mostly affects children in the preschool age. Numerous publications point out its natural, but also temporary, presence in the process of development, as long as the parents show patience and consistency [5,40,41]. Among the population of the children with ASD, food neophobia is part of food selectivity and is a frequent and serious problem that requires diagnostic and therapeutic attention [42]. Unfortunately, this problem does not resolve with time [11]. Although reluctance toward foods decreases gradually, and the disruptions in the mealtime behavior stop, the food repertoire undergoes the least significant fluctuations. Iimprovement can be achieved by applying nutritional therapy [41].

Parents use different methods in everyday life to encourage their children to consume an entire meal. These methods include verbal encouragement, directive verbal communication, positive or negative motivation, providing a variety of meals, giving a choice, engaging in food preparation, feeding the child, talking to the child, diverting the child’s attention or a parent’s own attitude during family mealtime. Parents of autistic children encourage their children to eat with the communication “If you eat, you will get” six times more frequently than the parents of healthy children, and they encourage their children by their own attitude and by organizing family mealtimes at the table three times less frequently than the parents of healthy children. The eating behaviors of healthy children are shaped significantly by the influence of their parents and the environment surrounding the meals. Encouraging the child to eat through incentives actually results in the acceptance and strengthening of the negative attitude of the child. Giving the child a choice between two meals creates a risk of an incorrect selection of the proposed food products, which should be similar in terms of the nutritional value. The best form of encouragement for a healthy child to eat is the proper attitude of the parent during family mealtimes, in the form of a feeling of satisfaction [43]. The improper attitude of the parent towards a child’s eating problems strengthens the improper patterns [13]. Nevertheless, based on our studies and observations, the behaviors, including the eating behaviors of autistic individuals, do not transfer by means of observation (which is, among others, the result of the disruption in the functioning of the mirror neurons and the outcomes of the deficit of the Theory of Mind).

One of the conditions for better appetite is to provide a sense of variety in meals by taking care of the appearance of the food. Parents, knowing their children, try to encourage them by paying attention to the way the food is presented (Table 14) [5,35].

Different scientists point out, in their publications, the role of eating as a form of social and cultural communication, which may, in some way, justify the autistic child’s problems related to functioning in this area. One way to improve mealtime behavior is to use an indispensable tool in the form of the help of a diagnostic and therapeutic team and to participate in nutritional training. Parents, feeling helpless, often ask for such support, and the ones who receive it report significant reduction in stress during mealtimes and increase in parental competencies [44,45]. It was indicated that parent knowledge, and improving parent competencies, in terms of supporting autistic individuals in eating is a significant factor of nutritional success [35].

An unexpected similarity between the groups arose concerning the indication of meals considered especially important for all family members, liked by all or prepared for special occasions for the majority of Respondents, these meals were valued similarly, showing that the social and cultural importance of eating mattered in both groups.

Feeding does not constitute just satisfying hunger but also preparing the meals with involvement, presenting the food in an esthetic and attractive way, as well as consuming the meals together with other family members, in a relaxed environment. The abovementioned principles are fundamental for healthy ways of eating, for the child and the entire family, as they facilitate the shaping and the development of proper nutritional behaviors and habits.

To summarize, the questionnaire we conducted indicated that feeding and eating for autistic children is often challenging for the parents, especially due to food selectivity frequently occurring in this group. Nevertheless, analyzing the entire questionnaire, it would be difficult not to realize that the two groups are similar to each other in many respects. The fact that not many differences were found resulted probably from the fact that in our study we had children with pure ASD, with greater acceptance for eating, with high functioning autism, and with Asperger’s Syndrome (AS) [28].

## 5. Limitations of the Study

The presented research is a pilot study presenting preliminary results. The objective was to identify endophenotypes among the patients with pure autism, including those related to feeding and eating. The authors used only their own questionnaire (Supplementary Questionnaire) which has not been validated. The results may be ambiguous due to the retrospective character of the questionnaire. Some of the questions are rather vague which results from the fact that the questionnaire itself is vast and the researchers did not want to overwhelm the parents with too many questions. Nevertheless, this causes difficulty in interpreting some of the results. Furthermore, it needs to be pointed out that the analysis was conducted on a rather small group of patients with a broad range of ages, between 2 and 12 years, which makes the interpretation of the collected data difficult. Also, despite the large volume of the questionnaire, only a handful of results indicated statistical significance, which may suggesta need to improve this scientific tool. This shows that caution should be used when formulating conclusions, and the study should be continued based on a revised questionnaire.

## 6. Conclusions

Feeding and eating problems should be considered on a wider scale than they have been, so far, in therapeutic programs, which should be extended to include the provision of psychological consultations and the facilitation of nutrition programs.

Food selectivity occurs significantly more frequently among children with ASD. Furthermore, parents introduce restrictive diets, such as the dairy-free diet and the gluten-free diet, on their own; therefore, the therapeutic team, assembled for the treatment of autism spectrum disorder, should include a gastroenterologists, having an important role, along with a closely cooperating experienced nutritionist.

## Figures and Tables

**Table 1:** Distribution of gender in the Study Group and the Control Group.

Gender	Study Group (*n* = 41; 100%)	Control Group (*n* = 34; 100%)	Fisher’s Exact Probability Test
Female	13 (31.7%)	8 (23.5%)	NS (*p* = 0.30)
Male	28 (68.3%)	26 (76.5%)	

**Table 2:** Distribution of age in the Study Group and the Control Group.

Age [in Years]	Study Group (*n* = 41; 100%)	Control Group (*n* = 34; 100%)	Chi-Square Test For Association with Yates Continuity Correction
from 3 to 7	18 (43.9%)	16 (47.1%)	NS (*p* = 0.73)
from 7 to 10	13 (31.7%)	8 (23.5%)	
above 10	10 (24.4%)	10 (29.4%)	
**Sex**	**Median** **(Years)**	**IQR** **(Years)**	**U Mann–Whitney** **Test**
Study Group (*n* = 41; 100%)	7.5	4.0	NS (*p* = 0.47)
Control Group (*n* = 34; 100%)	7.3	5.5	

**Table 3:** Distribution of appetite of children in the Study Group and the Control Group (question II/6).

Child’s Appetite	Study Group (*n* = 41; 100%)	Control Group (*n* = 34; 100%)	Fisher’s Exact Probability Test
Normal	19 (46.3%)	22 (64.7%)	*p* = 0.05
Reduced	17 (41.5%)	7 (20.6%)	
**Child’s Appetite**	**Study Group** **(*n* = 41; 100%)**	**Control Group** **(*n* = 34; 100%)**	**Fisher’s Exact Probability Test**
Normal + Increased	23 (56.1%)	26 (76.5%)	*p* = 0.05
Reduced	17 (41.5%)	7 (20.6%)	

**Table 4:** Distribution of fussing while consuming meals by children in the Study Group and the Control Group (question II/7).

Fussing	Study Group (*n* = 41; 100%)	Control Group (*n* = 34; 100%)	Fisher’s Exact Probability Test
Yes	24 (58.5%)	10 (29.4%)	*p* = 0.01
No	17 (41.5%)	24 (70.6%)	

**Table 5:** Distribution of entertaining activities during meal consumption for children in the Study Group and the Control Group (question II/8).

Entertaining	Study Group (*n* = 41; 100%)	Control Group (*n* = 34; 100%)	Fisher’s Exact Probability Test
Yes	17 (41.5%)	3 (8.8%)	*p* = 0.001
No	24 (58.5%)	31 (91.2%)	

**Table 6:** Method of consuming food by children in the Study Group and the Control Group (question II/11).

Method of Consuming Food	Study Group (*n* = 41; 100%)	Control Group (*n* = 34; 100%)	Fisher’s Exact Probability Test
Sitting at the table	25 (61%)	32 (94.1%)	*p* = 0.7777
Standing	3 (7.3%)	0	NS (*p* = 0.16)
Walking to the table	9 (22.0%)	2 (5.9%)	NS (*p* = 0.15)
Sitting on the floor	4 (9.8%)	0	*p* = 0.05

**Table 7:** What is used by the children in the Study Group and the Control Group to eat food (question II/12).

Method of Eating	Study Group (*n* = 41; 100%)	Control Group (*n* = 34; 100%)	Fisher’s Exact Probability Test
Spoon	36 (85.4%)	34 (100%)	*p* = 0.02
Fork	31 (75.6%)	34 (100%)	*p* = 0.001
Own hands	18 (43.9%)	11 (32.4%)	NS (*p* = 0.22)
Fed by parent	22 (53.7%)	5 (14.7%)	*p* = 0.0004

**Table 8:** Food selectivity in children in the Study Group and the Control Group (question II/13).

Food Selectivity	Study Group (*n* = 41; 100%)	Control Group (*n* = 34; 100%)	Fisher’s Exact Probability Test
Yes	26 (63.4%)	9 (26.5%)	*p* = 0.002
No	15 (36.4%)	25 (73.5%)	

**Table 9:** Tastes of food preferred by the children in the Study Group and the Control Group (question II/14).

Preferred Taste	Study Group (*n* = 41; 100%)	Control Group (*n* = 34; 100%)	Fisher’s Exact Probability Test
Sweet	24 (58.4%)	27 (79.4%)	*p* = 0.05
Salty	9 (22.0%)	7 (20.6%)	NS (*p* = 0.56)
Sour	5 (12.2%)	5 (14.7%)	NS (*p* = 0.51)
Bitter	0	0	---
Absence of taste preferences	13 (31.7%)	6 (17.7%)	NS (*p* = 0.13)
Child shows tendency to consume atypical things	3 (7.3%)	0	NS (*p* = 0.16)

**Table 10:** Textures of food preferred by the children in the Study Group and the Control Group (question II/15).

Texture of Food	Study Group (*n* = 41; 100%)	Control Group (*n* = 34; 100%)	Fisher’s Exact Probability Test
Fluid	9 (22.0%)	2 (5.9%)	*p* = 0.05
Semi-fluid, lumpy	5 (12.2%)	1 (3.3%)	NS (*p* = 0.15)
Minced/chopped food	8 (19.5%)	3 (8.8%)	NS (*p* = 0.17)
Solid food	15 (36.6%)	10 (29.4%)	NS (*p* = 0.34)
Indifferent to the texture of food	17 (41.5%)	21 (61.8%)	NS (*p* = 0.06)

**Table 11:** Who decides about the selection of foods consumed by the children in the Study Group and the Control Group (question II/19).

Who Decides	Study Group (*n* = 41; 100%)	Control Group (*n* = 34; 100%)	Fisher’s Exact Probability Test
Parent/Caregiver	29 (70.7%)	30 (88.2%)	NS (*p* = 0.06)
Child	17 (41.5%)	7 (20.6%)	*p* = 0.04
Nutritionist	0	0	---

**Table 12:** Types of restrictive diets used by the children in the Study Group and the Control Group (question II/20).

Restrictive Diet	Study Group (*n* = 41; 100%)	Control Group (*n* = 34; 100%)	Fisher’s Exact Probability Test
Dairy-free	5 (12.2%)	0	*p* = 0.04
Lactose-free	3 (7.3%)	1 (2.9%)	NS (*p* = 0.38)
Gluten-free	4 (9.8%)	0	NS (*p* = 0.08)
Egg-free	1 (2.4%)	1 (2.9%)	NS (*p* = 0.70)
Hypoallergenic	0	1 (2.9%)	NS (*p* = 0.45)
Elemental	0	0	---
Oligoantigenic	0	0	---
Vegetarian	0	0	---
Vegan	0	0	---
Rotation	0	0	---
Candida	1 (2.4%)	0	NS (*p* = 0.55)
macrobiotic	0	0	---
Other, please describe…	3 (7.3%) [sugar-free; apple-free]	1 (2.9%) [sugar-free]	NS (*p* = 0.38)

**Table 13:** Grading scale in selecting meals/products (provided in: mean; standard deviation; median) (question A/1).

Category	Study Group (*n* = 41)	Control Group (*n* = 34)	Mann-Whitney U Test
Color	3.2; 3.3; 2	2.6; 2.4; 2	NS (*p* = 0.84)
Shape	2.3; 3.2; 1	1.5; 1.9; 1	NS (*p* = 0.63)
Fragrance	5.7; 3.4; 7	4.8; 3.3; 5	NS (*p* = 0.25)
Taste	7.1; 3.4; 8	5.6; 3.7; 7	NS (*p* = 0.07)
Texture	5.2; 3.7; 5	2.6; 2.4; 2	*p* = 0.003
Other	(*n* = 7) 9.3; 1.1; 10	(*n* = 2) 8.5; 0.7; 8.5	---

**Table 14:** The method of convincing child in the Study Group and Control Group to eat (question A/9).

Method of Convincing	Study Group (*n* = 41; 100%)	Control Group (*n* = 34; 100%)	Fisher’s Exact Probability Test
Motivating: verbal motivating communication, e.g., “Eat a little more” or “Here comes the plane”.	6 (14.6%)	7 (20.6%)	NS (*p* = 0.35)
Directive: verbal communication, e.g., “Eat!”	8 (19.5%)	3 (8.8%)	NS (*p* = 0.17)
Motivating: “If you eat, you will get…,” etc.	18 (43.9%)	7 (20.6%)	*p* = 0.03
Motivating: “If you don’t eat, you will not go…,” etc.	5 (12.2%)	3 (8.8%)	NS (*p* = 0.47
Own attitude: by sitting at the table together	21 (51.2%)	27 (79.4%)	*p* = 0.01
Presenting food in a variety of ways: e.g., special plates, straws, or meals presented as play	9 (22.0%)	2 (5.9%)	*p* = 0.05
Feeding the child	7 (17.1%)	2 (5.9%)	NS (*p* = 0.13)
Engaging the child in preparation of food	8 (19.5%)	9 (26.5%)	NS (*p* = 0.33)
Talking to the child and using tricks	2 (4.9%)	2 (5.9%)	NS (*p* = 0.62)
Making the child watch TV to feed it	5 (12.2%)	2 (5.9%)	NS (*p* = 0.30)
Giving choice, e.g., “Would you like to eat cereal or a sandwich?”	22 (53.7%)	23 (67.7%)	NS (*p* = 0.16)

**Table 15:** Does the child eat food when the family eats at the table? (Question A/10).

Does the Child Eat More Willingly	Study Group (*n* = 40; 100%)	Control Group (*n* = 34; 100%)	Chi-Square Test for Association with Yates Continuity Correction
Yes	14 (35.0%)	22 (64.7%)	*p* = 0.04
No	5 (12.5%)	1 (2.9%)	
Difficult to say	21 (52.5%)	11 (32.4%)	
It does not matter if child is sitting together with the family or alone.			

**Table 16:** Parent’s self-efficacy scale on using devices during family mealtimes (provided in: mean; standard deviation; median) (question A/11).

Study Group (*n* = 41)	Control Group (*n* = 34)	Mann-Whitney U Test
3.1; 3.0; 2	1.7; 2.1; 1	*p* = 0.04

**Table 17:** Distribution of Parent’s self-efficacy scale scoring on using devices during family mealtimes question A/11).

Self-Efficacy Score	Study Group (*n* = 41)	Control Group (*n* = 34)	Chi-Square Test for Association with Yates Continuity Correction
0	10 (24.4%)	13 (38.2%)	*p* = 0.02
1–4	18 (43.9%)	19 (55.9%)	
5–10	13 (31.7%)	2 (5.9%)	

## Data Availability

The datasets generated during and/or analyzed during the current study are available from the corresponding author on reasonable request.

## Supplementary Materials

The following are available online at https://www.mdpi.com/article/10.3390/nu13113850/s1, Supplementary Questionnaire: QUESTIONNAIRE on EATING HABITS.
