# Supplementary material for: Eating Behaviors of Children with Autism—Pilot Study, Part II
Source: Nutrients. 2021 Oct 28;13(11):3850. doi: 10.3390/nu13113850 (PMC8620859; doi:10.3390/nu13113850)
Supplement: Supplementary file 1 [file nutrients-13-03850-s001.zip › nutrients-1425805-supplementary.pdf]

Own Questionnaire

QUESTIONNAIRE on EATING HABITS

The questionnaire on eating habits has been developed for the purpose of a thorough evaluation of the child's eating habits.
Part I relates to the child's eating habits within the 1st year of life (20 questions).
Part II relates to the child's current eating habits (20 questions).
An additional questionnaire, developed by an educational scientist and available in the attachment, relates to the eating habits of the child and its family (12 questions). The majority of the questions are closed-ended questions, to which the respondents will have yes (Y) or no (N) answers to choose from. Some questions are multiple choice questions, to which the respondents will be able to choose and mark the right answer (X). The rest of the questions are open questions, to which the respondents will be able to provide their own descriptive answer.
The tests are anonymous and will be used solely for the purposes related to a research study.
The Researches are available to answer any questions or concerns.

Child personal information:

1. Gender:

2. Date of birth:

3. Date of filling out this questionnaire:

PART II. Child's current eating habits

1. Please mark (X) the average mealtime duration:

a. Up to 10 minutes ( )

b. 10 to 20 minutes ( )

c. 20 – 30 minutes ( )

d. Above 30 minutes ( )

2. Please mark (X) any of the following words to describe the child's eating behavior:

a. Eats with pleasure ( )

b. Is indifferent towards food ( )

c. Needs to be persuaded to eat ( )

3. Does the child eat anything at night? Y / N

If the answer is yes, please mark (X) any of the following examples:

a. Milk ( )

b. Grits ( )

c. Sweets ( )

d. Bread ( )

e. Other ( ), please describe ____________________

4. Please mark (X) the quantity of snacks (food between the main, regular meals) consumed by the child during the day:

a. 1 ( )

b. 2 ( )

c. 3 ( )

d. 4 or more ( )

5. Please mark (X) the type of snack most frequently consumed by the child:

a. Fruit ( )

b. Vegetables ( )

c. Bread ( )

d. Sweets ( )

e. Salty snacks (chips, salted pretzels, crackers) ( )

f. Other ( ), please indicate the type _____________

6. Please mark (X) any of the following words to describe the child's appetite:

a. Normal ( )

b. Increased ( )

c. Decreased ( )

d. Lack of appetite ( )

7. Is the child fussy during the mealtime? Y / N

8. Does the child require any entertaining to divert it’s attention from the food during the mealtime? Y / N

9. Does the child consume meals with the family, as much as it is possible (i.e. with the other members of the family present) ? Y / N

10. Does the child consume meals regularly / at the same time every day? Y / N

11. Please mark (X) the position in which the child consumes meals:

a. Sitting at the table ( )

b. Standing ( )

c. Walking to the table ( )

d. Sitting on the floor ( )

12. Please mark (X) the way the child consumes meals:

a. Using a spoon ( )

b. Using a fork ( )

c. Using own hands ( )

d. Is fed by the caregiver ( )

13. Does the child express food selectiveness which means that the child eats only certain, selected type of products? Y / N

If the answer is yes, please mark (X) the products selected by the child:

a. Milk and dairy products ( )

b. Bread ( )

c. Meat products ( )

d. Fruit ( )

e. Sweets ( )

f. Other ( ), please describe ________________________

14. Please mark (X) the taste preferred by the child:

a. Sweet ( )

b. Salty ( )

c. Sour ( )

d. Bitter ( )

e. The child shows interest in consuming atypical or inedible things ( ). Please indicate ______________________________

15. Please mark (X) the preferred texture of the meals consumed by the child:

a. Fluid ( )

b. Semi-fluid, lumpy ( )

c. Minced / chopped food ( )

d. Solid products ( )

e. Indifferent to the texture of food ( )

16. Please mark (X) the preferred temperature of the food consumed by the child:

a. Hot food ( )

b. Cold food ( )

c. Optimal temperature for the particular type of food ( )

17. Please mark (X) the preferred fragrance of the food consumed by the child:

a. Very intensive, aromatic ( )

b. Subtle ( )

c. Fragrance-free ( )

d. Indifferent to fragrance ( )

18. Please mark (X) the type of food that the child does not like to eat:

a. Milk and dairy products ( )

b. Bread ( )

c. Meat products ( )

d. Eggs ( )

e. Fish ( )

f. Vegetables ( )

g. Fruit ( )

h. Sweets ( )

i. Processed food ( )

j. Other ( ), please indicate the type ___________________

k. Lack of any disliked foods ( )

19. Please place the mark (X) by the person who mainly decides about the food the child will consume:

a. Parent / caregiver ( )

b. Child ( )

c. Nutritionist ( )

20. Is the child currently on any restrictive diet? Y / N

If the answer is yes, please mark (X) the type of the diet:

a. Dairy-free ( )

b. Lactose-free ( )

c. Gluten-free ( )

d. Egg-free ( )

e. Hypoallergenic ( )

f. Elemental ( )

g. Oligoantigenic ( )

h. Vegetarian ( )

i. Vegan ( )

j. Rotation ( )

k. Candida diet ( )

l. Microbiotic diet ( )

m. Other type ( ), please describe ________________________________

ATTACHMENT. ADDITIONAL QUESTIONNAIRE FOR THE ASSESSMENT OF EATING HABITS, DEVELOPED BY AN EDUCATIONAL SCIENTIST.

1. In your opinion, what drives the child to choose the food / products that is acceptable to the child? On a scale of 1 to 10, please mark how much the child is driven by a given category: 0 means not at all, and 10 means a lot. Indicate the color, shape, fragrance, taste, texture, and/or other, mostly preferred by the child.

COLOR

Not at all A lot

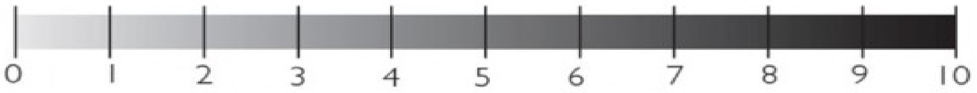

Preferred color ____

SHAPE

Not at all A lot

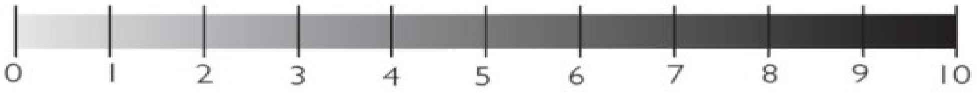

Preferred shape____

FRAGRANCE

Not at all A lot

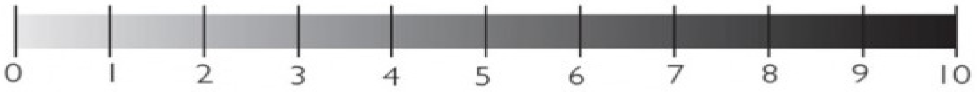

Preferred fragrance ____

TASTE

Not at all A lot

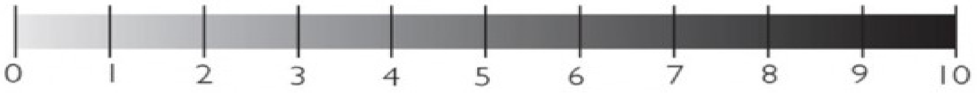

Preferred taste ____

TEXTURE

Not at all A lot

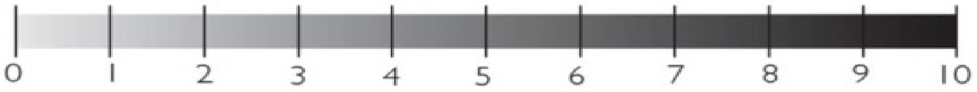

Preferred texture ____

OTHER

Not at all A lot

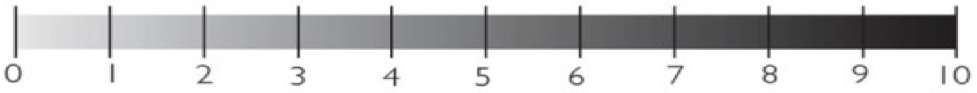

Preferred type ____

2. Is the child open to try new foods?

- Yes
- No
- No opinion

3. By which of the following were the parents motivated when feeding the child in the first months of the child's life?

- Child was fed on cue
- Child was fed according to a strict schedule
- I do not remember

4. How old was the child when the caregiving parent returned to work after the child's birth. Please mark the scale:

Did not go back to work | Went back to work after 36th month

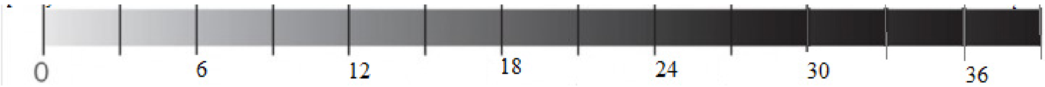

5. Who stayed with the child when the parents went back to work?

- Close family member
- Nanny
- Caregivers at the daycare center
- Caregivers at preschool

6. Do you have a table in the kitchen / dining room?

- Yes
- No

7. How often do you have family mealtimes? The following scale indicates a period of a week. Please mark the number of family mealtimes during a week on the scale. The beginning of the scale indicates zero family mealtimes, and the end of the scale indicates above 27 mealtimes.

Lack of family mealtimes | Above 27 family mealtimes during the week

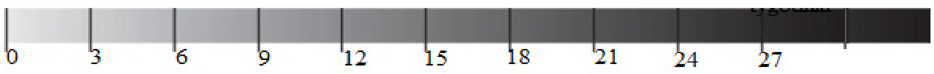

8. Which meal do you consume together most frequently?

- Breakfast
- Lunch
- Dinner
- Afternoon snack
- Supper
- Dinner / supper

9. How do you motivate the child to eat? (Mark up to 3 answers)

- Verbal encouragement, e.g.: please eat a little more…. Or: here comes the airplane…
- Directive verbal communication, e.g.: eat!
- Motivating: if you eat this, you will get / go … etc.
- Motivating: if you don't eat this, you will not get / go … etc.
- Setting a good example by organizing family mealtimes
- Presenting food in a variety of ways, e.g.: special plates, straws, interesting presentation of food on the plates
- Feeding the child
- Engaging the child in food preparation
- Distracting the child by talking to the child and tricking the child into eating the food
- Making the child watch TV in order to feed the child.
- Providing the child with a choice, e.g.: would you like to eat cereal or a sandwich?

10. Does the child eat the food more willingly with the whole family present at the table?

- Yes

- No

- It is difficult to say. It does not matter whether the family is present at the table or whether the child eats alone.

11. Do you have a habit of reading a newspaper, watching TV, listening to the radio or useing any other high technology devices during family mealtimes? Please mark the scale. Zero indicates that you never use such devices, and 10 indicates that you use such devices very often.

We never use any devices We use devices very often

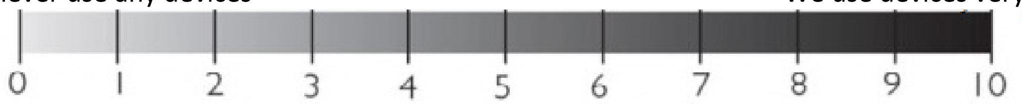

12. Is there any dish in your menu that is especially important for all members of your family which is enjoyed by everyone and/or is prepared on special occasions? If the answer is yes, please indicate that kind of dish.

- Yes

____
____

- No
